# Machine Learning Prediction Model for Dyslipidemia and Its Association With Atherothrombotic Events in 3 Independent Cohorts From South Korea, Japan, and the United Kingdom: Algorithm Development and Validation Study

**DOI:** 10.2196/81130

**Published:** 2026-05-19

**Authors:** Tae Hyeon Kim, Soeun Kim, Yerim Kim, Hayeon Lee, Seung Ha Hwang, So Young Yang, Lee Smith, André Hajek, Selin Woo, Dong Keon Yon

**Affiliations:** 1 Center for Digital Health, Medical Science Research Institute, Kyung Hee University Medical Center, Kyung Hee University College of Medicine, 23 Kyungheedae-ro, Dongdaemun-gu, Seoul, 02447, Republic of Korea, 82 2-961-0680, 82 2-961-0680; 2 Department of Precision Medicine, Kyung Hee University College of Medicine, Seoul, Republic of Korea; 3 Department of Computer Science, Sookmyung Women’s University, Seoul, Republic of Korea; 4 Department of Electronics and Information Convergence Engineering, Kyung Hee University, Yongin, Republic of Korea; 5 Cardiovascular Center, Kyung Hee University Hospital at Gangdong, Kyung Hee University School of Medicine, Seoul, Republic of Korea; 6 Department of Cardiology, Gil Medical Center, Gacheon University, Incheon, Republic of Korea; 7 Centre for Health Performance and Wellbeing, Anglia Ruskin University, Cambridge, United Kingdom; 8 Department of Public Health, Faculty of Medicine, Biruni University, Istanbul, Turkey; 9 Department of Health Economics and Health Services Research, University Medical Center Hamburg-Eppendorf, Hamburg Center for Health Economics, Hamburg, Germany; 10 Department of Pediatrics, Kyung Hee University Medical Center, Kyung Hee University College of Medicine, Seoul, Republic of Korea

**Keywords:** atherothrombotic events, dyslipidemia, machine learning, mortality, South Korea, Japan, United Kingdom

## Abstract

**Background:**

Dyslipidemia is a multifactorial and complex condition that warrants investigation through advanced analytical approaches such as machine learning (ML). Few previous ML studies predicting dyslipidemia have been validated across multiple international populations.

**Objective:**

This study aimed to develop an ML model to predict the 5-year incidence of dyslipidemia using routinely collected health examination data. To ensure generalizability, the model was externally validated in populations from South Korea, Japan, and the United Kingdom. Furthermore, the clinical relevance of the model-derived risk was evaluated by examining its association with atherosclerotic outcomes, including acute myocardial infarction and cerebral infarction.

**Methods:**

This study was conducted using 3 independent, large-scale, population-based cohorts. The discovery cohort from South Korea (n=471,650) was used for model training and internal validation, while 2 validation cohorts from Japan (validation A; n=7,255,685) and the United Kingdom (validation B; n=408,725) were used for external validation. We evaluated various ML-based models using 23 features extracted from regular health screening data to predict the new onset of dyslipidemia within 5 years. Shapley Additive Explanations values were calculated to assess feature importance. To ensure the robustness of the proposed ML model, we evaluated the risk of atherothrombotic events (acute myocardial infarction or cerebral infarction) based on the model probability (tertiles; T1, T2, and T3) using a Cox proportional hazards model.

**Results:**

In the discovery cohort, soft-voting ensemble learning with Light Gradient Boosting Machine and categorical boosting exhibited performance metrics of area under the receiver operating characteristic curve (AUROC) of 0.783, precision of 37.9%, and area under the precision-recall curve of 0.469. The model showed moderate discriminatory performance in the external validation cohorts (cohort A: AUROC 0.744; precision 27.2%; and cohort B: AUROC 0.687; precision 5.07%). Shapley Additive Explanations value analysis identified smoking, alcohol intake, and physical activity as the most important features for predicting dyslipidemia. Finally, a higher model probability (T3 vs reference) was pronounced with an increased risk of acute myocardial infarction (adjusted hazard ratio 2.34, 95% CI 1.84‐2.97) and cerebral infarction (adjusted hazard ratio 2.43, 95% CI 2.19‐2.71).

**Conclusions:**

This multinational study developed and validated an ML-based model using routine health checkup data to predict the 5-year risk of new-onset dyslipidemia, which was also associated with atherosclerotic events.

## Introduction

Dyslipidemia is defined as an abnormal lipid profile, characterized by elevated low-density lipoprotein (LDL) cholesterol or triglycerides or reduced high-density lipoprotein (HDL) cholesterol [1]. This condition is recognized as a significant contributor to the development of numerous health issues, most notably cardiovascular disease [2]. Given its chronic nature, effective management is essential to mitigate associated health risks and reduce the overall health care burden [3]. While dyslipidemia can be diagnosed simply through blood tests, predicting an individual’s future risk of developing the condition is challenging. Accurate risk prediction could enable proactive early intervention, thereby controlling its burden. Consequently, there has been increasing interest in using machine learning (ML) to forecast the incidence of dyslipidemia.

However, ML models from previous studies for predicting dyslipidemia have several limitations. Many models focus on single populations, leading to insufficient validation across diverse cohorts [4]. Some models rely on uncommon features, such as genetic markers [5], or predict only specific forms of dyslipidemia, such as familial hypercholesterolemia [6]. Such genetic markers are not readily accessible for widespread application, and dyslipidemia can be diagnosed based on variations in any component of the lipid profile [7]. Consequently, the risk of complications persists regardless of the specific type of lipid abnormality, underscoring the limitations of previous models [8].

Therefore, this study aimed to develop an ML model for predicting the incidence of dyslipidemia within 5 years using readily available routine health examination data. To enhance generalizability, the model was validated across multiple populations in South Korea, Japan, and the United Kingdom. Additionally, we examined whether the model-derived dyslipidemia risk score was associated with subsequent atherothrombotic events as an interpretive analysis of clinical plausibility and alignment with established risk patterns.

## Methods

### Overview

In this study, we developed and validated an ML model to predict dyslipidemia within 5 years based on the initial health screening data from the Korean National Health Insurance Service–National Sample Cohort (NHIS-NSC). Model development was conducted using the Korean cohort, followed by external validation using 2 independent populations: the Japan Medical Data Center (JMDC) cohort in Japan and the UK Biobank in the United Kingdom. This multicohort approach enabled robust assessment of model generalizability across diverse health care systems [9]. To enhance interpretability, we applied explainable artificial intelligence (AI) techniques, including Shapley Additive Explanations (SHAP), to identify key contributing features and elucidate the decision-making process of the models [10]. Additionally, we used Cox proportional hazards regression using the predicted probabilities from the models to assess the longitudinal risk of atherothrombotic events, such as acute myocardial infarction and cerebral infarction, thereby showing the broader clinical plausibility of our framework. This study was conducted and reported in accordance with established Transparent Reporting of a Multivariable Prediction Model for Individual Prognosis or Diagnosis (TRIPOD)–AI guidelines for prediction model research (Table S1 in Multimedia Appendix 1).

### Data Sources

We used 3 large-scale, population-based cohort datasets to develop and validate an ML model for predicting dyslipidemia. The primary cohort was derived from the NHIS-NSC of South Korea (n=1,062,018), a claim-based cohort. Two external validation cohorts were subsequently used: the JMDC dataset (validation cohort A; n=21,517,570), also a claim-based cohort; and the UK Biobank (validation cohort B; n=502,367), a large-scale population-based prospective cohort [9]. All data used in the analyses were fully deidentified and anonymized prior to access, in compliance with relevant ethical and data protection regulations.

### Discovery Cohort

The NHIS-NSC includes 1,062,018 individuals who underwent nationwide health screening between January 1, 2009, and December 31, 2013. This time frame was selected because several predictor variables became newly available and were consistently recorded starting in 2009, and December 31, 2017, was designated as the end point for follow-up. This cohort served as the discovery dataset for development and internal validation of an ML model designed to predict the occurrence of dyslipidemia within 5 years after each participant’s baseline health screening. Dyslipidemia was defined based on the presence of *International Classification of Diseases (ICD), 10th revision*, codes E78 in administrative claims data. The ICD-10 system is a globally standardized classification framework developed by the World Health Organization (WHO), ensuring consistent diagnostic coding across countries and health care systems [11]. The index date was designated as the date of each participant’s first health screening during the enrollment period. Participants without a dyslipidemia diagnosis during follow-up, including those with less than 5 years of follow-up, were classified as noncases.

### Data Preprocessing

**Figure 1.**
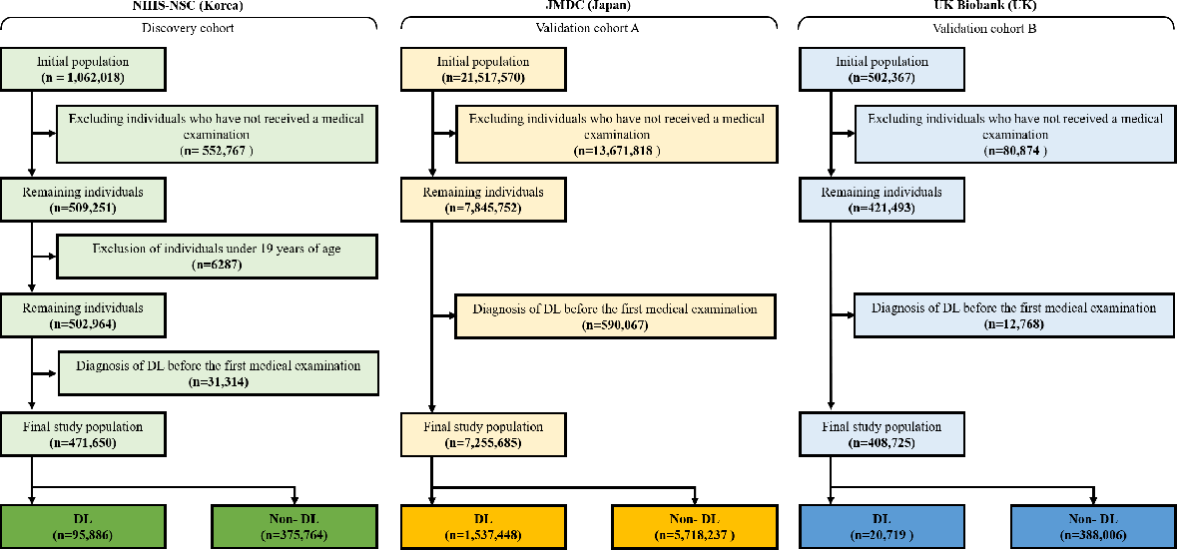
Flow diagram of participant selection for the algorithm development and validation cohort study evaluating machine learning–based dyslipidemia prediction. The diagram shows inclusion and exclusion processes applied to adults enrolled in the National Health Insurance Service–National Sample Cohort (NHIS-NSC; South Korea), Japan Medical Data Center (JMDC) claims database (Japan), and UK Biobank (the United Kingdom). DL: dyslipidemia.

As illustrated in Figure 1, we applied the following exclusion criteria: (1) individuals who did not undergo health screening during the study period; (2) individuals younger than 19 years; and (3) individuals with a prior diagnosis of dyslipidemia before their index date, defined as the date of their first health screening. The dataset was split into training and testing sets (7:3) using stratified sampling based on the incident dyslipidemia outcome variable, and all preprocessing steps were fitted on the training data and applied to the test data. Continuous variables with missing values were imputed using the K-nearest neighbors (KNN) algorithm fitted on the training data and applied to the test data [12]. Categorical variables were not imputed; missing values were treated as a separate “unknown” category. These values were handled as an explicit category rather than as missing data and were retained in the analysis. To address class imbalance, the Synthetic Minority Over-Sampling Technique was applied exclusively within each training fold during cross-validation to prevent information leakage [9]. Synthetic Minority Over-Sampling Technique generates synthetic samples of the minority class by interpolating between existing minority instances, thereby increasing sample diversity without altering the original distribution of the validation or test sets. Finally, all input features were normalized using Min-Max scaling to ensure comparability across variables [10]. Min-Max scaling transforms each feature to a fixed range, typically [0,1], while preserving the original distribution of the data.

### Predictor Variables

A total of 23 features were used to predict dyslipidemia within 5 years, encompassing demographic, socioeconomic, clinical, and lifestyle factors. In the demographic and socioeconomic category, sex (male and female), age (years; continuous), region of residence (urban and rural), and household income (low: 0th‐39th percentile, middle: 40th‐79th percentile, and high: 80th‐100th percentile) were included [9]. In the external validation cohorts, income variables were harmonized into this predefined 3-level structure prior to model input to ensure structural consistency across datasets. Clinical measures comprised LDL cholesterol, HDL cholesterol, total cholesterol, triglycerides, aspartate aminotransferase, alanine aminotransferase, γ-glutamyl transpeptidase, waist circumference, systolic blood pressure, diastolic blood pressure, fasting blood glucose, hemoglobin, and BMI. Additionally, lifestyle and medical history factors encompassed smoking status (never, ex, and current); alcohol intake (never, 1‐2 d/wk, 3‐4 d/wk, and ≥5 d/wk); physical activity (never, 1‐2 d/wk, 3‐4 d/wk, and ≥5 d/wk); and relevant comorbidities, such as a history of hypertension, diabetes, or stroke (Table 1). For variables with structurally different formats across cohorts, predefined mapping rules were applied to ensure consistency. For example, binary physical activity variables were mapped by assigning “no” to “never” and “yes” to ≥1 session per week. Detailed mapping rules are provided in Table S2 in Multimedia Appendix 1.

**Table 1.** Baseline demographic, clinical, and laboratory characteristics of adults included in the model development cohort from the NHIS-NSC^a^ in South Korea at baseline.

Characteristics	Total (N=471,650)	Dyslipidemia (n=95,886)	Nondyslipidemia (n=375,764)
Age (y), mean (SD)	51.04 (23.04)	58.24 (12.99)	49.16 (14.85)
Sex, n (%)			
Male	238,706 (50.61)	45,592 (47.55)	193,114 (51.39)
Female	232,944 (49.39)	50,294 (52.45)	182,650 (48.61)
Region of residence, n (%)			
Urban	275,448 (58.40)	55,205 (57.57)	220,243 (58.61)
Rural	196,202 (41.60)	40,681 (42.43)	155,521 (41.39)
Household income, n (%)			
Low	141,206 (29.94)	27,685 (28.87)	113,521 (30.21)
Middle	200,686 (42.55)	38,125 (39.76)	162,561 (43.26)
High	123,840 (26.26)	29,037 (30.28)	94,803 (25.23)
Unknown	5918 (1.25)	1039 (1.08)	4879 (1.30)
Smoking status, n (%)			
Never smoker	288,112 (61.09)	61,056 (63.68)	227,056 (60.43)
Ex-smoker	60,874 (12.91)	14,410 (15.03)	46,464 (12.37)
Current smoker	115,808 (24.55)	18,884 (19.69)	96,924 (25.79)
Unknown	6856 (1.45)	1536 (1.60)	5320 (1.42)
Alcohol intake (d/wk), n (%)			
Never	345,135 (73.18)	71,948 (75.03)	273,187 (72.70)
1‐2	58,789 (12.46)	9876 (10.30)	48,913 (13.02)
3‐4	34,248 (7.26)	6524 (6.80)	27,724 (7.38)
≥5	11,164 (2.37)	2305 (2.40)	8859 (2.36)
Unknown	22,314 (4.73)	5233 (5.46)	17,081 (4.55)
Physical activity (sessions per week), n (%)			
Never	360,649 (76.47)	71,298 (74.36)	289,351 (77.00)
1‐2	69,681 (14.77)	14,401 (15.02)	55,280 (14.71)
3‐4	23,701 (5.03)	5611 (5.85)	18,090 (4.81)
≥5	5347 (1.13)	1362 (1.42)	3985 (1.06)
Unknown	12,272 (2.60)	3214 (3.35)	9058 (2.41)
History of hypertension, n (%)	64,980 (13.78)	26,430 (27.56)	38,550 (10.26)
History of diabetes, n (%)	23,131 (4.90)	9898 (10.32)	13,233 (3.52)
History of stroke, n (%)	4394 (0.93)	1400 (1.46)	2994 (0.80)
Systolic blood pressure (mm Hg), mean (SD)	122.07 (15.28)	126.24 (15.85)	121.01 (14.95)
Diastolic blood pressure (mm Hg), mean (SD)	76.02 (10.21)	78.19 (10.45)	75.47 (10.07)
Fasting blood glucose (mg/dL), mean (SD)	97.42 (24.18)	104.01 (31.21)	95.73 (21.71)
Total cholesterol (mg/dL), mean (SD)	194.87 (36.84)	211.14 (42.97)	190.72 (33.87)
HDL^b^ cholesterol (mg/dL), mean (SD)	56.53 (28.36)	55.46 (31.38)	56.80 (27.53)
LDL^c^ cholesterol (mg/dL), mean (SD)	113.79 (37.47)	125.35 (43.56)	110.86 (35.15)
Hemoglobin (g/dL), mean (SD)	13.88 (1.64)	13.85 (1.56)	13.89 (1.66)
Triglycerides (mg/dL), mean (SD)	131.13 (93.41)	162.64 (113.56)	123.10 (85.70)
γ-glutamyl transpeptidase (U/L), mean (SD)	36.36 (51.88)	43.22 (59.52)	34.61 (49.59)
Aspartate aminotransferase (U/L), mean (SD)	25.17 (17.06)	27.31 (19.69)	24.62 (16.28)
Alanine aminotransferase (U/L), mean (SD)	24.71 (21.99)	28.11 (24.50)	23.84 (21.22)
BMI (kg/m^2^), mean (SD)	23.63 (3.30)	24.57 (3.16)	23.39 (3.29)
Waist circumference (cm), mean (SD)	79.87 (9.31)	82.71 (8.76)	79.15 (9.30)

a NHIS-NSC: National Health Insurance Service–National Sample Cohort.

b HDL: high-density lipoprotein.

c LDL: low-density lipoprotein.

### External Validation Dataset

External validation cohort A (JMDC) included individuals in Japan who underwent health screenings between January 1, 2006, and June 30, 2024. External validation cohort B (UK Biobank) comprised participants from the United Kingdom who enrolled in this prospective cohort between January 1, 2006, and April 30, 2023. For both external cohorts, the prediction target was incident dyslipidemia occurring within 5 years after each participant’s baseline health screening, consistent with the discovery cohort. In cohort A, dyslipidemia was defined using the same ICD-10 criteria as in the discovery cohort. In external validation cohort B, dyslipidemia was defined using both ICD-10 and ICD-9 codes (ICD-9: 272) to ensure compatibility with historical records. Consistently, the same exclusion criteria applied to the discovery cohort were used for both validation cohorts, as illustrated in Figure 1 and Tables S3 and S4 in Multimedia Appendix 1. Missing values were imputed using the KNN method, consistent with the preprocessing applied to the discovery dataset [12]. Additionally, all input features in the validation datasets were normalized using the same Min-Max scaling method applied to the training data to ensure consistency in feature distribution.

### Model Development and Validation

To predict dyslipidemia within 5 years, we developed and evaluated multiple ML algorithms, including logistic regression, random forest, extreme gradient boosting (XGBoost), adaptive boosting (AdaBoost), Light Gradient Boosting Machine (LightGBM), and categorical boosting (CatBoost) [10,13]. To ensure robust and generalizable model performance, 5-fold cross-validation was used on the training set derived from the discovery cohort during model development. In this approach, the dataset was randomly partitioned into 5 equally sized folds, with each fold serving once as a validation set, while the remaining 4 were used for training. This strategy enabled evaluation across diverse subsamples, thereby reducing the risk of overfitting and improving generalizability. The optimal classification threshold was determined using Youden’s J statistic calculated across the 5 cross-validation folds in the discovery cohort only. The final threshold, defined as the mean optimal value across folds, was fixed prior to external validation and subsequently applied unchanged to the internal test set and both external validation cohorts to preserve independence and avoid data leakage. To further enhance overall predictive performance, we performed grid search–based hyperparameter tuning within a cross-validation framework to identify optimal model configurations [10]. During this process, key hyperparameters were tuned using an internal validation split, and an early stopping criterion was applied to prevent overfitting. Among the models tested, a soft-voting ensemble combining CatBoost and LightGBM was selected to improve robustness across heterogeneous multinational datasets by leveraging the complementary strengths of both algorithms [14]. The final optimized hyperparameters for both models are summarized in Table S5 in Multimedia Appendix 1 to facilitate reproducibility. After identifying the optimal hyperparameters through cross-validation, the final model was retrained on the entire training dataset using these parameters, rather than using an ensemble of cross-validation models.

The final models trained on the discovery cohort’s training set were subsequently evaluated on the internal test set, as well as on 2 external validation cohorts A and B. This evaluation framework allowed us to assess the generalizability of the models across distinct populations and health care systems. To ensure consistency across cohorts, all predictor variables were harmonized according to the definitions used in the discovery cohort. Model performance was assessed using several metrics, including the area under the receiver operating characteristic curve (AUROC), precision, sensitivity, specificity, accuracy, *F*_1_-score, and the area under the precision-recall curve (AUPRC). Given the class imbalance, precision and AUROC were emphasized as the primary evaluation metrics [10,15]. To obtain 95% CIs for performance metrics, cross-validation procedures were applied in the training dataset. CIs were not reported for the validation and independent test datasets, as these were evaluated a single time to preserve their role in unbiased model assessment.

### Sensitivity Analysis

Two sensitivity analyses were conducted to assess the robustness of the modeling strategy. First, a model excluding baseline lipid measures (LDL cholesterol, HDL cholesterol, triglycerides, and total cholesterol) was developed to evaluate whether model performance was driven by lipid biomarkers directly related to the outcome definition, yielding similar discrimination to the main model. Second, a complete-case sensitivity analysis was additionally conducted by refitting the discovery cohort model using only participants without missing values to assess the impact of imputation. These results collectively support the stability and validity of the preprocessing and modeling procedures.

### Calibration and Decision Curve Analysis

Model calibration was evaluated in each cohort by estimating calibration-in-the-large, calibration slope, and the Brier score. Calibration-in-the-large was calculated as the difference between the mean predicted probability and the observed event rate, while calibration slope was estimated by regressing observed outcomes on the logit-transformed predicted probabilities. The Brier score was used to quantify overall prediction error [11]. Calibration plots were generated by comparing observed versus predicted risks across deciles of predicted probability. To assess potential improvements in transportability, simple recalibration was explored by adjusting the model intercept and slope in each external validation cohort while preserving the original predictor coefficients. Discrimination and calibration metrics before and after recalibration were compared to evaluate the impact of cohort-specific recalibration.

Clinical plausibility was further examined using decision curve analysis, which estimates the net benefit of the prediction model across a range of clinically plausible threshold probabilities [11]. Net benefit was compared with default strategies of treating all individuals or treating none, using the predicted 5-year dyslipidemia risk.

### Explainable AI

To improve model transparency and interpretability, we applied SHAP values, which quantify the marginal contribution of each feature to individual predictions [10,16]. SHAP values allow both global and local interpretation: the distribution of SHAP values across the population reveals not only the relative importance of features but also the direction of their influence (ie, whether higher values of a feature increase or decrease dyslipidemia risk). Additionally, the average magnitude of SHAP values helps identify the most influential predictors overall, while individual-level SHAP analyses enable a step-by-step understanding of how specific features shape a single prediction [16]. This explainable AI approach transforms the model from a black box into a clinically interpretable tool, highlighting consistent and actionable risk factors. All processes were performed using Python (version 3.11.4; Python Software Foundation). Key libraries from our toolbox included Scikit-learn (version 1.2.2; Scikit-learn development team), NumPy (version 1.24.1; Python Software Foundation), and Pandas (version 1.5.3; Python Software Foundation) for ML tasks and data wrangling.

### Statistical Analysis

To evaluate the broader clinical plausibility of the final ML model, the predicted 5-year dyslipidemia probability was used as the primary exposure to assess the risk of diseases with increased incidence associated with dyslipidemia. We acknowledge that associations between the predicted risk score and atherothrombotic outcomes may reflect shared underlying cardiovascular risk factors included in the prediction model. A binary classification threshold, determined using Youden’s J statistic during 5-fold cross-validation in the discovery cohort, was applied to define incident dyslipidemia. Individuals with predicted probabilities below this threshold were used as the reference group. Among individuals with predicted probabilities equal to or above the threshold, predicted risks were further categorized into 3 tertiles (T1, T2, and T3). The primary outcomes were acute myocardial infarction (ICD-10: I21; and ICD-9: 410) and cerebral infarction (ICD-10: I63; and ICD-9: 434.01, 434.11). To assess the risk of these outcomes based on predicted dyslipidemia probability, we applied a Cox proportional hazards regression model to estimate adjusted hazard ratios (aHRs) and 95% CIs. The Cox models were sequentially adjusted as follows: model 1 adjusted for age and sex (primary minimally confounded estimate); and model 2 adjusted for age, sex, region, and income (conservative estimate adjusted for key sociodemographic factors). To account for competing risks, we conducted sensitivity analyses using Fine–Gray subdistribution hazard models for acute myocardial infarction and cerebral infarction, treating death as a competing event. Statistical significance was defined as a 2-sided *P* value <.05. All statistical analyses were performed using SAS software (version 9.4; SAS Institute Inc).

### Ethical Considerations

The study protocol was approved by the Institutional Review Board of Kyung Hee University. Documentation of the institutional review board approval is provided in Figure S1 in Multimedia Appendix 1. The requirement for informed consent was waived in accordance with relevant ethical guidelines for secondary use of administrative data. All datasets used in this study were fully anonymized or deidentified prior to analysis, and no personally identifiable information was accessible to the investigators. No financial or other compensation was provided to participants for this study. The manuscript and all supplementary materials do not contain any images or information that could enable the identification of individual participants. All studies were conducted following the ethical guidelines and regulations of their respective countries.

### Deployment Governance

A model card was developed to document the model’s intended purpose, target population, input features, outputs, and key limitations, including circumstances in which the tool should not be used. The web-based app is intended solely for research and educational purposes and is not designed for clinical decision-making, diagnosis, or self-management. User inputs are processed in real time and are not retained at the individual level, ensuring that no personally identifiable information is stored or recoverable.

To support responsible deployment, a governance framework has been established for postdeployment oversight. The system does not store or log individual-level input data; however, it may record anonymized and aggregated summary statistics (eg, distributions of input features and model outputs) that cannot be linked to any individual user. These aggregated metrics are used exclusively to monitor potential data drift, performance drift, and subgroup-level variation. Model performance and calibration are periodically reviewed, with updates planned on a quarterly basis to maintain reliability and transparency.

## Results

### Study Population

Among the 1,062,018 participants initially included in the discovery cohort from South Korea, 552,767 individuals who had not undergone regular health screenings and 6287 individuals aged <19 years were excluded. Furthermore, 31,314 individuals with a history of dyslipidemia diagnosis prior to their first health screening were also excluded, resulting in a final discovery cohort of 471,650 participants. Among these participants, 95,886 (20.33%) were identified as patients with dyslipidemia (Figure 1 and Table 1). Variable-wise missingness for the discovery cohorts is summarized in Table S6 in Multimedia Appendix 1, providing an overview of the distribution of incomplete data prior to preprocessing. Similar exclusion criteria were applied to the validation cohorts, resulting in 7,255,685 participants in validation cohort A from Japan and 408,725 participants in validation cohort B from the United Kingdom (Figure 1 and Tables S3 and S4 in Multimedia Appendix 1).

### Model Training and Validation

Table 2 presents the results of 5-fold cross-validation of various ML models trained on the discovery cohort from South Korea. LightGBM and CatBoost showed superior performance as single models, particularly regarding AUROC and precision. To further enhance prediction performance, we developed an ensemble model using a soft-voting approach with LightGBM and CatBoost. Detailed hyperparameter configurations for both algorithms are provided in Table S5 in Multimedia Appendix 1 to facilitate reproducibility. This ensemble model exhibited improved performance metrics: AUROC, 0.782 (95% CI 0.782‐0.783); precision, 37.6% (95% CI 37.0‐38.1); sensitivity, 74.2% (95% CI 73.0%‐75.5%); specificity, 68.5% (95% CI 67.2‐69.8); accuracy, 69.6% (95% CI 68.8‐70.4); balanced accuracy, 71.4% (95% CI 71.2‐71.5); *F*_1_-score, 49.9% (95% CI 49.6‐50.1); and AUPRC, 0.464 (95% CI 0.462‐0.466).

**Table 2.** A comparative analysis of the predictive performance of models using training and test datasets from South Korea and external validation datasets from Japan and the United Kingdom.

Model	AUROC^a^	Precision	Sensitivity	Specificity	Accuracy	Balanced accuracy	*F*_1_-score	AUPRC^b^
Train dataset (South Korea; 5-fold cross-validation), mean (95% CI)^c^								
Logistic regression	0.762 (0.761‐0.763)	35.9 (35.5‐36.3)	74.3 (72.8‐75.9)	66.1 (64.9‐67.4)	67.8 (67.1‐68.5)	70.2 (70.1‐70.4)	48.4 (48.3‐48.5)	0.411 (0.409‐0.413)
Random forest	0.769 (0.768‐0.770)	35.8 (35.3‐36.2)	75.1 (74.0‐76.3)	65.5 (64.4‐66.7)	67.5 (66.8‐68.2)	70.3 (70.3‐70.4)	48.5 (48.3‐48.6)	0.441 (0.438‐0.444)
XGBoost^d^	0.778 (0.776‐0.779)	37.3 (36.0‐38.5)	74.2 (71.6‐76.8)	68.0 (65.3‐70.7)	69.3 (67.6‐70.9)	71.1 (71.0‐71.3)	49.6 (49.1‐50.1)	0.459 (0.457‐0.462)
AdaBoost^e^	0.781 (0.780‐0.781)	37.6 (37.3‐37.9)	74.2 (73.7‐74.7)	68.5 (68.0‐69.1)	69.7 (69.3‐70.0)	71.4 (71.2‐71.5)	49.9 (49.7‐50.1)	0.462 (0.459‐0.465)
LightGBM^f^	0.782 (0.780‐0.784)	37.7 (37.1‐38.3)	74.6 (73.7‐75.6)	68.5 (67.2‐69.8)	69.7 (68.9‐70.6)	71.6 (71.4‐71.7)	50.1 (49.7‐50.4)	0.472 (0.470‐0.474)
CatBoost^g^	0.781 (0.781‐0.782)	36.1 (35.8‐36.5)	74.5 (73.9‐75.1)	66.4 (65.6‐67.2)	68.0 (67.5‐68.5)	70.4 (70.3‐70.6)	48.7 (48.4‐48.9)	0.443 (0.441‐0.445)
CatBoost+LightGBM^h^^,^^i^	0.782 (0.782‐0.783)	37.6 (37.0‐38.1)	74.2 (73.0‐75.5)	68.5 (67.2‐69.8)	69.6 (68.8‐70.4)	71.4 (71.2‐71.5)	49.9 (49.6‐50.1)	0.464 (0.462‐0.466)
Test dataset (South Korea)^j^								
Logistic regression	0.763	35.6	76.1	64.8	67.1	70.4	48.5	0.413
Random forest	0.770	36.0	74.6	66.2	67.9	70.4	48.6	0.445
XGBoost	0.779	37.3	74.6	67.9	69.3	71.3	49.7	0.462
AdaBoost	0.783	37.6	74.7	68.4	69.7	71.6	50.1	0.467
LightGBM	0.782	38.7	73.5	70.3	70.9	71.9	50.7	0.475
CatBoost	0.783	37.8	71.7	69.8	70.2	70.8	49.5	0.446
CatBoost+LightGBM^i^	0.783	37.9	74.1	69.0	70.1	71.6	50.2	0.469
External validation dataset (Japan)^j^								
CatBoost+LightGBM^i^	0.744	27.2	81.9	40.9	49.6	61.4	40.8	0.302
External validation dataset (UK)^j^								
CatBoost+LightGBM^i^	0.687	5.07	79.5	22.7	25.6	51.1	9.65	0.245

a AUROC: area under the receiver operating characteristic curve.

b AUPRC: area under the precision-recall curve.

c CIs were calculated for the training dataset using cross-validation.

d XGBoost: extreme gradient boosting.

e AdaBoost: adaptive boosting.

f LightGBM: Light Gradient Boosting Machine.

g CatBoost: categorical boosting.

h GBM: gradient boosting machine.

i Model with the best performance.

j Validation and test datasets were evaluated once without CIs to preserve unbiased assessment.

The ensemble model combining LightGBM and CatBoost via a soft-voting approach also indicated the best performance on the test dataset of the discovery cohort from South Korea: AUROC, 0.783; precision, 37.9%; sensitivity, 74.1%; specificity, 69.0%; accuracy, 70.1%; balanced accuracy, 71.6%; *F*_1_-score, 50.2%; and AUPRC, 0.469. The similar performance between the cross-validation and test dataset results suggests minimal overfitting or underfitting of the model.

Performance of the proposed ensemble model on the external validation cohorts from Japan and the United Kingdom is also presented in Table 2. In external validation cohort A (Japan), the model achieved an AUROC of 0.744, a precision of 27.2%, a sensitivity of 81.9%, a specificity of 40.9%, an accuracy of 49.6%, a balanced accuracy of 61.4%, an *F*_1_-score of 40.8%, and an AUPRC of 0.302. In external validation cohort B (UK), the performance metrics were an AUROC of 0.687, a precision of 5.07%, a sensitivity of 79.5%, a specificity of 22.7%, an accuracy of 25.6%, a balanced accuracy of 51.1%, an *F*_1_-score of 9.65%, and an AUPRC of 0.245. Across cohorts from South Korea, Japan, and the United Kingdom, the proposed model showed broadly comparable discriminatory performance in predicting 5-year dyslipidemia incidence using regular health screening data, suggesting a degree of cross-population applicability.

### Sensitivity Analysis

As sensitivity analyses, we evaluated the robustness of the model to potential label leakage and missing data handling. First, the model was refitted in the Korean discovery cohort after excluding baseline lipid measurements (LDL cholesterol, HDL cholesterol, triglycerides, and total cholesterol). The lipid-free model showed slightly lower but broadly comparable discrimination to the main model (AUROC 0.767 vs 0.783), suggesting that although lipid biomarkers contributed to predictive performance, the overall model retained reasonable discrimination without them. Second, a complete-case analysis restricted to participants without missing values yielded discrimination similar to that of the imputed analysis (AUROC 0.785 vs 0.783), suggesting that KNN-based imputation did not materially affect model performance. Detailed results are provided in Table S7 in Multimedia Appendix 1.

### Calibration and Clinical Plausibility

Across cohorts, the prediction model showed acceptable calibration, with cohort-specific variation in calibration-in-the-large and slope (Table S8 in Multimedia Appendix 1). Calibration-in-the-large values were modestly negative across cohorts, indicating a tendency toward overestimation of absolute risk, while calibration slopes suggested mild overdispersion or underdispersion depending on the cohort. Brier scores remained within an acceptable range, reflecting stable overall predictive accuracy. Calibration plots illustrating observed versus predicted risks are presented in Figure S2 in Multimedia Appendix 1. In the UK cohort, the uncalibrated model showed systematic overestimation of absolute risk, indicating calibration drift. However, simple local recalibration using intercept and slope adjustment substantially improved calibration-in-the-large and calibration slope, while discrimination (AUROC) remained largely unchanged. Recalibration enhanced risk estimation without materially affecting ranking performance. Decision curve analysis showed that the model provided a positive net benefit across a range of clinically relevant threshold probabilities compared with treat-all and treat-none strategies (Figure S3 in Multimedia Appendix 1), supporting its potential utility for risk stratification in preventive care settings.

### SHAP Value Analysis

**Figure 2.**
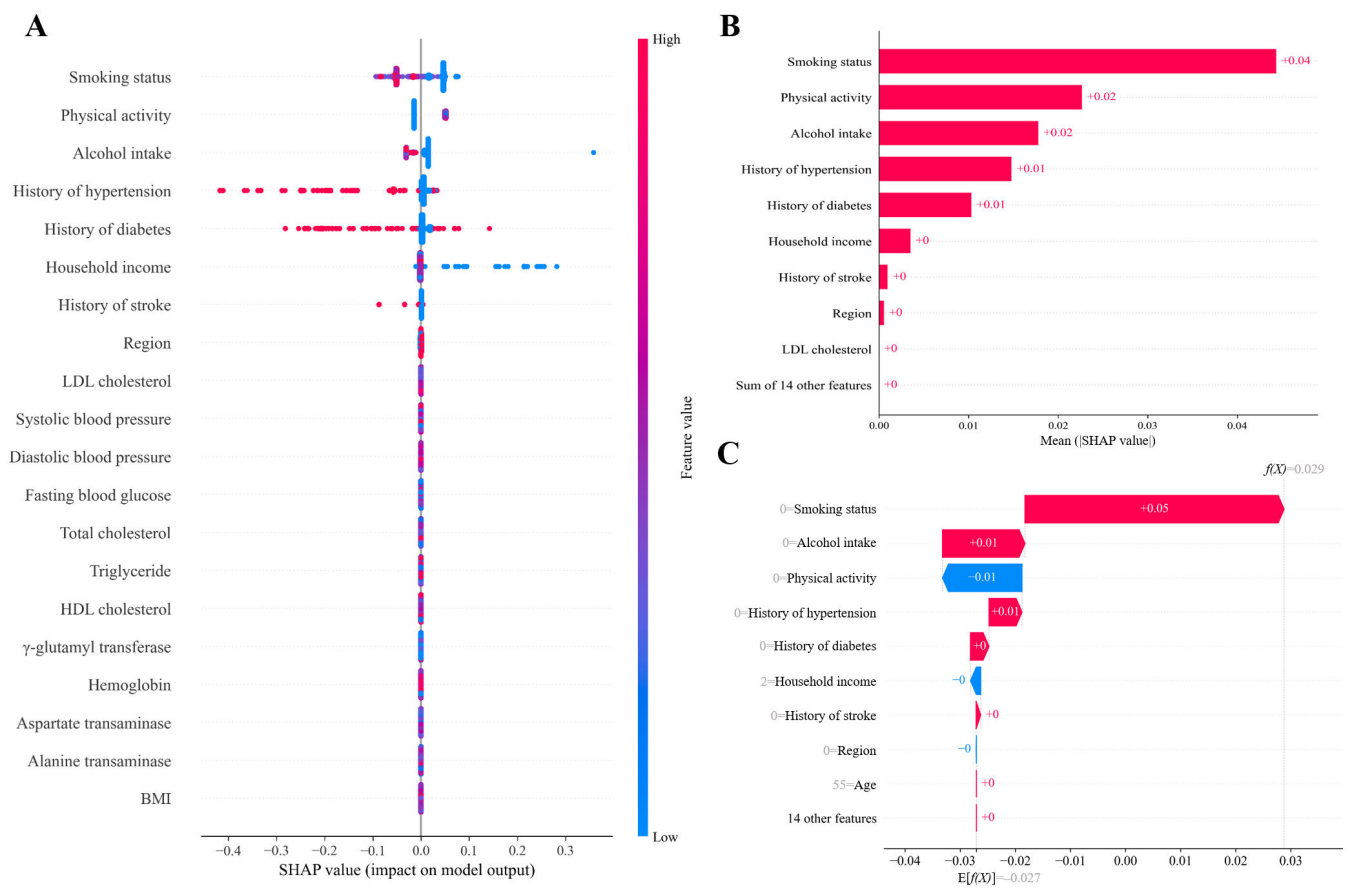
Shapley Additive Explanations (SHAP) analysis of feature contributions in the machine learning–based dyslipidemia prediction model. (A) Summary plot of SHAP values showing the impact and direction of each feature. (B) Mean absolute SHAP values across the cohort, identifying the most influential predictors. (C) SHAP decision plot for a representative individual. The consistency of key features across all panels highlights the robustness of model interpretation. HDL: high-density lipoprotein; LDL: low-density lipoprotein.

To examine the influence of features used to predict dyslipidemia within 5 years, SHAP values were calculated for the proposed ensemble model combining LightGBM and CatBoost via a soft-voting approach. In this model, smoking had the largest influence on predicting incident dyslipidemia, followed by physical activity and alcohol intake. Histories of hypertension, diabetes, and stroke were also found to be important predictors (Figure 2).

### Risk of Atherothrombotic Events Based on ML-Predicted Probability

Table 3 presents the risk of atherothrombotic events (acute myocardial infarction and cerebral infarction), evaluated based on the model probability in the discovery cohort from South Korea. For acute myocardial infarction, the risk increased significantly from the first tertile of predicted probability (T1: aHR 1.62, 95% CI 1.27‐2.06), with further increases observed in T2 (aHR 1.74, 95% CI 1.37‐2.19) and T3 (aHR 2.34, 95% CI 1.84‐2.97). The risk of cerebral infarction was significantly elevated across all tertiles, showing a graded association with increasing predicted probability (T1: aHR 1.59, 95% CI 1.42‐1.79; T2: aHR 2.16, 95% CI 1.94‐2.39; and T3: aHR 2.43, 95% CI 2.19‐2.71). These findings indicate a trend where higher predicted dyslipidemia probability was associated with an increased risk of these outcomes, for which dyslipidemia is a known risk factor. Results from the Fine–Gray subdistribution hazard models, accounting for death as a competing risk, are presented in Table S9 in Multimedia Appendix 1. Compared with the Cox proportional hazards models, the subdistribution hazard ratios were attenuated or reversed in direction, likely reflecting the influence of competing mortality risk in higher risk groups. This pattern suggests that individuals with higher predicted dyslipidemia risk may also have higher competing mortality risk, which can reduce the probability of observing the cardiovascular outcome in competing risk models.

**Table 3.** Atherothrombotic events according to tertiles of model probability.

Probability^a^	Model 1^b^, aHR^c^ (95% CI)	Model 2^d^, aHR (95% CI)
Acute myocardial infarction		
Less than threshold	1.0 (reference)	1.0 (reference)
T1	1.68 (1.32‐2.12)*^e^*	1.62 (1.27‐2.06)*^e^*
T2	1.80 (1.44‐2.25)^e^	1.74 (1.37‐2.19)^e^
T3	2.41 (1.93‐3.01)^e^	2.34 (1.84‐2.97)^e^
Cerebral infarction		
Less than threshold	1.0 (reference)	1.0 (reference)
T1	1.59 (1.41‐1.78)*^e^*	1.59 (1.42‐1.79*)^e^*
T2	2.13 (1.92‐2.37)*^e^*	2.16 (1.94‐2.39)*^e^*
T3	2.40 (2.16‐2.67)*^e^*	2.43 (2.19*‐*2.71)*^e^*

a The classification threshold was determined using Youden’s J statistic during 5-fold cross-validation in the discovery cohort.

b Model 1: adjusted for age and sex.

c aHR: adjusted hazard ratio.

d Model 2: adjusted for age, sex, region of residence (urban and rural), and household income (low: 0th‐39th percentile, middle: 40th‐79th percentile, and high: 80th‐100th percentile).

e Statistical significance (*P*<.05).

### AI-Driven Web Application

Our final model for predicting incident dyslipidemia within 5 years, based on regular health checkup data, is deployed on our website [17] (Figure S4 in Multimedia Appendix 1). The application collects 23 parameters from regular health screening data to evaluate the probability of incident dyslipidemia. The web application uses encrypted communication protocols (HTTPS), and user-entered information is processed transiently in server memory for real-time risk calculation and deleted immediately after generating results. No individual-level user inputs are stored, logged, or retained, and no personally identifiable information is collected. Aggregated and anonymized summary statistics may be recorded for system monitoring purposes. Access to the hosting environment is restricted to authorized administrators only, and the application is intended solely for research and educational purposes.

## Discussion

### Key Findings

This study investigated an ML-based approach for predicting dyslipidemia within 5 years using regular health screening data from 3 large-scale population-based cohorts with distinct characteristics (South Korea, Japan, and the United Kingdom). The soft-voting ensemble learning with LightGBM and CatBoost exhibited superior performance (AUROC 0.783 and precision 37.9%). This level of discrimination was observed across cohorts from South Korea, Japan, and the United Kingdom, suggesting that the model retained a degree of ranking ability across populations. Importantly, although discrimination remained acceptable across cohorts, calibration varied substantially, particularly in the UK cohort. Our findings suggest that local recalibration can effectively address this issue, underscoring that recalibration should be considered a necessary step prior to deployment in new health care settings. The proposed model identified smoking, physical activity, and alcohol intake as important features for predicting dyslipidemia incidence. In addition, individuals with higher model probabilities showed elevated risks of atherothrombotic events, including acute myocardial infarction and cerebral infarction.

### Plausible Mechanism

The prominent role of lifestyle factors, such as smoking status, physical activity, and alcohol intake, in our model’s prediction of incident dyslipidemia is consistent with their well-established impact on lipid metabolism. Smoking contributes to adverse lipid profiles through multiple mechanisms, including the promotion of systemic inflammation, increased oxidative stress, and the dysregulation of enzymes critical to lipoprotein metabolism and clearance [18]. These changes can lead to elevated levels of total cholesterol, LDL cholesterol, and triglycerides, along with reduced HDL cholesterol [19]. Excessive alcohol intake results in increased triglyceride accumulation in the liver and plasma, driven by impaired hepatic fatty acid oxidation and stimulated hepatic lipogenesis and very-low-density lipoprotein synthesis and secretion [20,21]. Conversely, regular physical activity favorably alters lipid profiles by enhancing lipoprotein lipase activity, which promotes efficient triglyceride clearance, and facilitating cholesterol removal from peripheral tissues [22].

Furthermore, the importance of pre-existing conditions, such as hypertension, diabetes, and a history of stroke, as predictors reflects their deep metabolic and pathological interconnections with dyslipidemia. Hypertension and type 2 diabetes, often components of the metabolic syndrome, share underlying mechanisms (eg, insulin resistance and systemic inflammation) that contribute to unfavorable lipid profiles and accelerated atherosclerosis [19,23]. A history of stroke, indicating established atherosclerosis and often poorly controlled risk factors, suggests ongoing metabolic derangements that predispose individuals to dyslipidemia [24]. Their inclusion as important predictors is biologically plausible, reflecting their established roles in complex metabolic and cardiovascular pathology.

### Comparisons With Previous Studies

Previous ML studies predicting dyslipidemia or related conditions have been conducted; however, they have important limitations [4-6,25-28]. These frequently included reliance on single populations with limited sample sizes [4], a focus on specific, narrow forms of dyslipidemia such as familial hypercholesterolemia rather than comprehensive profiles [6,25-27], or the incorporation of data not routinely available, such as genetic markers [5,26,28]. Consequently, their scalability and utility in widespread clinical practice are often constrained.

In contrast, this study developed an ML model using readily accessible clinical indicators derived from routine health screening data. We suggested the model’s generalizability through validation in large cohorts from 3 countries. Following an established framework for clinical prediction models [29], model development and validation were performed; however, clinical performance assessment has not yet been conducted, indicating the need for further research. Nevertheless, we observed that a higher predicted probability of incident dyslipidemia was associated with a higher risk of atherothrombotic events for which dyslipidemia is a risk factor. These findings suggest that the model-derived predictions of dyslipidemia are clinically valid, as they show a significant association with the occurrence of atherosclerotic events consistent with established clinical evidence.

### Policy Implications

Although dyslipidemia itself may not severely reduce quality of life or pose an immediate risk of death, its critical role as a precursor to atherosclerosis and subsequent occlusive vascular events, such as myocardial infarction and cerebral infarction, which can directly or indirectly lead to mortality, underscores the necessity of early diagnosis and management [30,31]. This necessity highlights the importance of developing effective strategies for early detection and targeted intervention. Accordingly, predictive modeling approaches for dyslipidemia are of significant interest for public health policy and clinical practice.

In response to this need for predictive tools, our study developed an ML model using readily available routine health screening data. This model exhibits the feasibility and clinical plausibility of predicting dyslipidemia within 5 years, enabling the identification of high-risk individuals for targeted early intervention to reduce future disease burden. From a policy perspective, this approach could support population-level risk stratification strategies by integrating model-derived risk scores into existing national health screening programs. Such integration may allow health systems to prioritize preventive counseling, lifestyle modification programs, and follow-up monitoring for individuals at high predicted risk, while avoiding unnecessary interventions in low-risk groups. In clinical guideline development, ML-based risk stratification could complement traditional lipid-based thresholds by incorporating broader cardiometabolic and lifestyle factors, thereby facilitating more personalized and resource-efficient prevention strategies. Furthermore, the use of routinely collected data enhances scalability and feasibility, making this framework particularly relevant for public health planning and implementation across diverse health care settings.

While lipid profile variables (total cholesterol, LDL cholesterol, HDL cholesterol, and triglycerides) were also included to predict the 5-year risk of dyslipidemia, their feature contribution in the model was not prominent. This contrasts with the significant importance of lifestyle factors, such as smoking, alcohol intake, and physical activity, identified by the model. This finding may underscore the necessity of continuous efforts toward lifestyle improvement, rather than complacency based on currently normal lipid profiles.

### Limitations

This study has several limitations. First, our reliance on data from regular health screenings meant that potentially important risk factors for dyslipidemia, such as diet, genetic factors, and family history, were not available. Nonetheless, the model’s ability to predict dyslipidemia using routinely collected variables supports its potential practicality for large-scale implementation. Second, although the prediction horizon was defined on an individual-specific 5-year basis, calendar time–based internal validation was not performed. In addition, participants without a recorded dyslipidemia diagnosis during follow-up, including those with less than 5 years of follow-up, were classified as noncases. This approach assumes that individuals with truncated follow-up would not have developed dyslipidemia within the full 5-year prediction window, potentially introducing outcome misclassification and censoring-related bias. Consequently, the model’s robustness to temporal changes in coding practices, reimbursement policies, or health care use patterns could not be fully evaluated. Third, missing values were handled using KNN imputation and Min-Max scaling; however, these distance-based methods may be sensitive to feature scaling and data distribution, and their influence on model performance cannot be fully excluded. As scaling was applied after KNN imputation, distance calculations during imputation may have been influenced by differences in feature scale. This ordering was chosen to preserve the original data distribution prior to transformation. All preprocessing was performed on the training data to minimize information leakage. Complete-case sensitivity analyses yielded similar results, suggesting that the overall impact is expected to be limited, although some uncertainty may remain. Fourth, dyslipidemia was defined using ICD-10 diagnostic codes extracted from administrative claims data, which may introduce outcome misclassification. Although laboratory measurements such as LDL cholesterol, HDL cholesterol, triglycerides, and total cholesterol were available, they were not incorporated into the outcome definition because longitudinal follow-up laboratory measurements were not consistently available across cohorts within the 5-year prediction window. In addition, laboratory thresholds and treatment practices differ across countries, reducing cross-country comparability. Using ICD-10 codes provided a standardized framework for outcome definition across Korea, Japan, and the United Kingdom, although differences in national screening practices and treatment thresholds may partly explain calibration differences across cohorts. Fifth, despite efforts to harmonize variables across countries, complete comparability cannot be guaranteed because of differences in health care systems, population characteristics, and data structures. While the model showed broadly comparable discriminatory performance across cohorts from South Korea, Japan, and the United Kingdom, precision was lower in the UK cohort. This likely reflects the substantially lower baseline prevalence of dyslipidemia in the United Kingdom dataset (approximately 5%) compared with the Korean and Japanese cohorts (approximately 20%), together with the use of a fixed classification threshold derived from the Korean discovery cohort. The threshold was fixed across all external validation cohorts to preserve model independence and avoid data-driven optimization. This lower prevalence may partly reflect the characteristics of the UK Biobank, a volunteer-based prospective cohort with a well-documented healthy participant selection bias, as well as potential underascertainment of dyslipidemia due to reliance on diagnostic coding [32]. In contrast, the Korean and Japanese cohorts were based on administrative claims data, which more comprehensively capture routinely managed conditions such as dyslipidemia. As precision is influenced by outcome prevalence, applying the same threshold to populations with different baseline risks may reduce positive predictive value. Cohort-specific recalibration or threshold adjustment may therefore be required before clinical deployment. Sixth, a lipid-free sensitivity analysis yielded a slightly lower AUROC than the full ensemble model (0.767 vs 0.783); however, the absolute difference in discrimination was modest and likely reflects a limited impact on overall model performance. This result should therefore be interpreted cautiously. Seventh, categorical variables were encoded using ordinal representations, which may impose a linear structure across categories. As a result, SHAP values reflect each variable as a single ordered feature and should be interpreted as overall trends rather than category-specific effects. Eighth, although Cox proportional hazards models were used to evaluate associations between predicted dyslipidemia risk and subsequent cardiovascular outcomes, these models estimate cause-specific hazards and do not fully account for competing risks such as death. Sensitivity analyses using Fine–Gray subdistribution hazard models were therefore conducted, treating death as a competing event. Differences between the Cox and Fine–Gray estimates likely reflect the influence of competing mortality risk in higher risk groups, as the 2 approaches quantify distinct epidemiological quantities. This may indicate that individuals with higher predicted dyslipidemia risk also have a greater burden of comorbidities or overall frailty, which could increase mortality before cardiovascular events are observed. Finally, given the observational nature of this study, the associations between predicted dyslipidemia risk and subsequent cardiovascular outcomes should not be interpreted as causal relationships.

Despite these limitations, this study shows the feasibility of using routinely collected health screening data from large-scale population-based cohorts across 3 countries to predict dyslipidemia within 5 years and to explore its potential association with atherothrombotic events.

### Conclusions

This study developed an ML-based model for predicting dyslipidemia within 5 years using regular health screening data. The proposed model was evaluated across 3 independent national cohorts (South Korea, Japan, and the United Kingdom). A higher model-derived probability of incident dyslipidemia was significantly associated with the occurrence of atherosclerotic events, indicating alignment with established clinical evidence and supporting its potential clinical applicability. Although the model shows promise, incorporating additional features and validating its application in more diverse populations may further enhance its utility.

## Supplementary material

10.2196/81130 Multimedia Appendix 1 Supplementary tables (S1–S9) and figures (S1–S4) supporting the main findings of this study.

## Abbreviations

AdaBoost: adaptive boosting
aHR: adjusted hazard ratio
AI: artificial intelligence
AUPRC: area under the precision-recall curve
AUROC: area under the receiver operating characteristic curve
CatBoost: categorical boosting
HDL: high-density lipoprotein
ICD: International Classification of Diseases
JMDC: Japan Medical Data Center
KNN: K-nearest neighbors
LDL: low-density lipoprotein
LightGBM: Light Gradient Boosting Machine
ML: machine learning
NHIS-NSC: Korean National Health Insurance Service–National Sample Cohort
SHAP: Shapley Additive Explanations
TRIPOD: Transparent Reporting of a Multivariable Prediction Model for Individual Prognosis or Diagnosis
WHO: World Health Organization
XGBoost: Extreme Gradient Boosting
