# Supplementary material for: Machine Learning Prediction Model for Dyslipidemia and Its Association With Atherothrombotic Events in 3 Independent Cohorts From South Korea, Japan, and the United Kingdom: Algorithm Development and Validation Study
Source: JMIR Med Inform. 2026 May 19;14:e81130. doi: 10.2196/81130 (PMC13186430; doi:10.2196/81130)

| **Supplementary Material** |
| --- |

Original Article

**Machine learning prediction model for dyslipidemia and its association with atherothrombotic events in three independent cohorts from South Korea, Japan, and the UK: an algorithm development and validation study**

**Running title:** Prediction for dyslipidemia

Tae Hyeon Kim^1,2&^, Soeun Kim^1,2&^, Yerim Kim^1,3^, Hayeon Lee^1,4^, Seung Ha Hwang^1,4^, So Young Yang^5,6^, Lee Smith^7,8^, André Hajek^9^, Selin Woo^1,2*^ Dong Keon Yon^1,2,10*^

^&^ These authors contributed equally to this work as first authors.

^*^ These authors jointly supervised this work.

**^*^Corresponding authors**

**Selin Woo**, PhD

Center for Digital Health, Medical Science Research Institute, Kyung Hee University Medical Center, Kyung Hee University College of Medicine, 23 Kyungheedae-ro, Dongdaemun-gu, Seoul 02447, Republic of Korea

E-mail: dntpfls@naver.com

**Dong Keon Yon**, MD, PhD, FAAAAI, FACAAI, ATSF (lead contact)

Center for Digital Health, Medical Science Research Institute, Kyung Hee University Medical Center, Kyung Hee University College of Medicine, 23 Kyungheedae-ro, Dongdaemun-gu, Seoul 02447, Republic of Korea

E-mail: yonkkang@gmail.com

**Table S1.** TRIPOD+AI checklist.

| Section/Topic | Item | Development / evaluation^*^ | Checklist item | Reported on page |
| --- | --- | --- | --- | --- |
| **TITLE** | | | | |
| *Title* | 1 | D;E | Identify the study as developing or evaluating the performance of a multivariable prediction model, the target population, and the outcome to be predicted | Title |
| **ABSTRACT** | | | | |
| *Abstract* | 2 | D;E | See TRIPOD+AI for Abstracts checklist | Abstract |
| **INTRODUCTION** | | | | |
| *Background* | 3a | D;E | Explain the healthcare context (including whether diagnostic or prognostic) and rationale for developing or evaluating the prediction model, including references to existing models | Introduction |
|  | 3b | D;E | Describe the target population and the intended purpose of the prediction model in the context of the care pathway, including its intended users (e.g., healthcare professionals, patients, public) | Introduction |
|  | 3c | D;E | Describe any known health inequalities between sociodemographic groups | Introduction |
| *Objectives* | 4 | D;E | Specify the study objectives, including whether the study describes the development or validation of a prediction model (or both) | Introduction |
| **METHODS** | | | | |
| *Data* | 5a | D;E | Describe the sources of data separately for the development and evaluation datasets (e.g., randomized trial, cohort, routine care or registry data), the rationale for using these data, and representativeness of the data | Data sources, Discovery cohort, External validation dataset |
|  | 5b | D;E | Specify the dates of the collected participant data, including start and end of participant accrual; and, if applicable, end of follow-up | Data sources, Discovery cohort, External validation dataset |
| *Participants* | 6a | D;E | Specify key elements of the study setting (e.g., primary care, secondary care, general population) including the number and location of centres | Data sources, Discovery cohort, External validation dataset |
|  | 6b | D;E | Describe the eligibility criteria for study participants | Data sources, Discovery cohort, External validation dataset |
|  | 6c | D;E | Give details of any treatments received, and how they were handled during model development or evaluation, if relevant | Data sources, Discovery cohort, External validation dataset |
| *Data preparation* | 7 | D;E | Describe any data pre-processing and quality checking, including whether this was similar across relevant sociodemographic groups | Data preprocessing |
| *Outcome* | 8a | D;E | Clearly define the outcome that is being predicted and the time horizon, including how and when assessed, the rationale for choosing this outcome, and whether the method of outcome assessment is consistent across sociodemographic groups | Discovery cohort, External validation dataset |
|  | 8b | D;E | If outcome assessment requires subjective interpretation, describe the qualifications and demographic characteristics of the outcome assessors | Discovery cohort, External validation dataset |
|  | 8c | D;E | Report any actions to blind assessment of the outcome to be predicted | Discovery cohort, External validation dataset |
| *Predictors* | 9a | D | Describe the choice of initial predictors (e.g., literature, previous models, all available predictors) and any pre-selection of predictors before model building | Predictor variables |
|  | 9b | D;E | Clearly define all predictors, including how and when they were measured (and any actions to blind assessment of predictors for the outcome and other predictors) | Predictor variables |
|  | 9c | D;E | If predictor measurement requires subjective interpretation, describe the qualifications and demographic characteristics of the predictor assessors | Predictor variables |
| *Sample size* | 10 | D;E | Explain how the study size was arrived at (separately for development and evaluation), and justify that the study size was sufficient to answer the research question. Include details of any sample size calculation | Data sources |
| *Missing data* | 11 | D;E | Describe how missing data were handled. Provide reasons for omitting any data | Data preprocessing |
| *Analytical methods* | 12a | D | Describe how the data were used (e.g., for development and evaluation of model performance) in the analysis, including whether the data were partitioned, considering any sample size requirements | Model development and validation |
|  | 12b | D | Depending on the type of model, describe how predictors were handled in the analyses (functional form, rescaling, transformation, or any standardisation) | Model development and validation |
|  | 12c | D | Specify the type of model, rationale^†^, all model-building steps, including any hyperparameter tuning, and method for internal validation | Model development and validation |
|  | 12d | D;E | Describe if and how any heterogeneity in estimates of model parameter values and model performance was handled and quantified across clusters (e.g., hospitals, countries). See TRIPOD-Cluster for additional considerations^‡^ | Model development and validation |
|  | 12e | D;E | Specify all measures and plots used (and their rationale) to evaluate model performance (e.g., discrimination, calibration, clinical utility) and, if relevant, to compare multiple models | Model development and validation |
|  | 12f | E | Describe any model updating (e.g., recalibration) arising from the model evaluation, either overall or for particular sociodemographic groups or settings | Model development and validation |
|  | 12g | E | For model evaluation, describe how the model predictions were calculated (e.g., formula, code, object, application programming interface) | Model development and validation |
| *Class imbalance* | 13 | D;E | If class imbalance methods were used, state why and how this was done, and any subsequent methods to recalibrate the model or the model predictions | Model development and validation |
| *Fairness* | 14 | D;E | Describe any approaches that were used to address model fairness and their rationale | NA |
| *Model output* | 15 | D | Specify the output of the prediction model (e.g., probabilities, classification). Provide details and rationale for any classification and how the thresholds were identified | Model development and validation |
| *Training versus evaluation* | 16 | D;E | Identify any differences between the development and evaluation data in healthcare setting, eligibility criteria, outcome, and predictors | Model development and validation |
| *Ethical approval* | 17 | D;E | Name the institutional research board or ethics committee that approved the study and describe the participant-informed consent or the ethics committee waiver of informed consent | Ethical statement |
| **OPEN SCIENCE** | | | | |
| *Funding* | 18a | D;E | Give the source of funding and the role of the funders for the present study | Footnote |
| *Conflicts of interest* | 18b | D;E | Declare any conflicts of interest and financial disclosures for all authors | Footnote |
| *Protocol* | 18c | D;E | Indicate where the study protocol can be accessed or state that a protocol was not prepared | Footnote |
| *Registration* | 18d | D;E | Provide registration information for the study, including register name and registration number, or state that the study was not registered | Footnote |
| *Data sharing* | 18e | D;E | Provide details of the availability of the study data | Footnote |
| *Code sharing* | 18f | D;E | Provide details of the availability of the analytical code^§^ | Footnote |
| **PATIENT & PUBLIC INVOLVEMENT** | | | | |
| *Patient & Public Involvement* | 19 | D;E | Provide details of any patient and public involvement during the design, conduct, reporting, interpretation, or dissemination of the study or state no involvement | Footnote |
| **RESULTS** | | | | |
| *Participants* | 20a | D;E | Describe the flow of participants through the study, including the number of participants with and without the outcome and, if applicable, a summary of the follow-up time. A diagram may be helpful | Study population |
|  | 20b | D;E | Report the characteristics overall and, where applicable, for each data source or setting, including the key dates, key predictors (including demographics), treatments received, sample size, number of outcome events, follow-up time, and amount of missing data. A table may be helpful. Report any differences across key demographic groups | Study population |
|  | 20c | E | For model evaluation, show a comparison with the development data of the distribution of important predictors (demographics, predictors, and outcome) | Study population |
| *Model development* | 21 | D;E | Specify the number of participants and outcome events in each analysis (e.g., for model development, hyperparameter tuning, model evaluation) | Model training and validation |
| *Model specification* | 22 | D | Provide details of the full prediction model (e.g., formula, code, object, application programming interface) to allow predictions in new individuals and to enable third-party evaluation and implementation, including any restrictions to access or re-use (e.g., freely available, proprietary)^‖^ | Model training and validation |
| *Model performance* | 23a | D;E | Report model performance estimates with confidence intervals, including for any key subgroups (e.g., sociodemographic). Consider plots to aid presentation | Model training and validation |
|  | 23b | D;E | If examined, report results of any heterogeneity in model performance across clusters. See TRIPOD Cluster for additional details^‡^ | Model training and validation |
| *Model updating* | 24 | E | Report the results from any model updating, including the updated model and subsequent performance | Model training and validation |
| **DISCUSSION** | | | | |
| *Interpretation* | 25 | D;E | Give an overall interpretation of the main results, including issues of fairness in the context of the objectives and previous studies | Key findings |
| *Limitations* | 26 | D;E | Discuss any limitations of the study (such as a non-representative sample, sample size, overfitting, missing data) and their effects on any biases, statistical uncertainty, and generalizability | Limitations |
| *Usability of the model in the context of current care* | 27a | D | Describe how poor quality or unavailable input data (e.g., predictor values) should be assessed and handled when implementing the prediction model | Comparison of previous studies, Policy implication |
|  | 27b | D | Specify whether users will be required to interact in the handling of the input data or use of the model, and what level of expertise is required of users | Comparison of previous studies, Policy implication |
|  | 27c | D;E | Discuss any next steps for future research, with a specific view to applicability and generalizability of the model | Comparison of previous studies, Policy implication |

*Abbreviations*: NA, not applicable.

^*^ D=items relevant only to the development of a prediction model; E=items relating solely to the evaluation of a prediction model; D;E=items applicable to both the development and evaluation of a prediction model

^†^ Separately for all model building approaches

^‡^ TRIPOD-Cluster is a checklist of reporting recommendations for studies developing or validating models that explicitly account for clustering or explore heterogeneity in model performance (eg, at different hospitals or centres). Debray et al, BMJ 2023; 380: e071018 [DOI: 10.1136/bmj-2022-071018]

^§^ This relates to the analysis code, for example, any data cleaning, feature engineering, model building, evaluation

^‖^ This relates to the code to implement the model to get estimates of risk for a new individual

^¶^ Reference: Collins GS, Moons KGM, Dhiman P, et al. BMJ 2024;385:e078378. doi:10.1136/bmj-2023-078378

**Table S2.** Variable harmonization scheme and mapping rules for aligning external cohorts to the discovery cohort.

| **Variable** | **External cohort category** | **Mapped category (Discovery cohort)** |
| --- | --- | --- |
| **Physical activity** | |  |
|  | No | Never |
|  | Yes | ≥1 session per week |
| **Alcohol intake** | |  |
|  | Rarely | Never |
|  | Sometimes | 1–4 days per week |
|  | Every day | ≥5 days per week |
| **Household income** | |  |
|  | Q1 | Low |
|  | Q2–Q4 | Middle |
|  | Q5 | High |

Q1–Q5 represent cohort-specific income quantiles prior to harmonization.

**Table S3.** Baseline characteristics in Japan.

|  | **Total (n=7,255,685)** | **Dyslipidemia (n=1,537,448)** | **Non-dyslipidemia (n=5,718,237)** |
| --- | --- | --- | --- |
| **Mean age (SD), *y*** | 41.94 (11.70) | 46.90 (10.56) | 40.61 (11.63) |
| **Sex, *n (%)*** |  |  |  |
| Male | 4,294,791 (59.19) | 976,439 (63.51) | 3,318,352 (58.03) |
| Female | 2,960,894 (40.81) | 561,009 (36.49) | 2,399,885 (41.97) |
| **Smoking status, *n (%)*** |  |  |  |
| No | 1,704,362 (23.49) | 374,395 (24.35) | 1,329,967 (23.26) |
| Yes | 5,099,651 (70.28) | 1,069,343 (69.55) | 4,030,308 (70.48) |
| Unknown | 451,672 (6.23) | 93,710 (6.10) | 357,962 (6.26) |
| **Alcohol intake (days per week), n (%)** |  |  |  |
| Rarely | 2,544,110 (35.06) | 536,139 (34.87) | 2,007,971 (35.12) |
| Sometimes | 2,444,681 (33.69) | 477,350 (31.05) | 1,967,331 (34.40) |
| Every day | 1,349,973 (18.61) | 328,672 (21.38) | 1,021,301 (17.86) |
| Unknown | 916,921 (12.64) | 195,287 (12.70) | 721,634 (12.62) |
| **Physical activity (sessions per week), n (%)** |  |  |  |
| No | 4,916,696 (67.76) | 1,037,416 (67.48) | 3,879,280 (67.84) |
| Yes | 1,276,791 (17.60) | 279,380 (18.17) | 997,411 (17.44) |
| Unknown | 1,062,198 (14.64) | 220,652 (14.35) | 841,546 (14.72) |
| **History of hypertension, *n (%)*** | 418,649 (5.77) | 207,279 (13.48) | 211,370 (3.70) |
| **History of diabetes, *n (%)*** | 95,322 (1.31) | 58,858 (3.83) | 36,464 (0.64) |
| **History of stroke, *n (%)*** | 51,352 (0.71) | 8,505 (0.55) | 42,847 (0.75) |
| **Systolic blood pressure, mmHg, mean (SD)** | 118.48 (15.92) | 123.14 (17.19) | 117.23 (15.33) |
| **Diastolic blood pressure, mmHg, mean (SD)** | 72.84 (11.81) | 76.68 (12.44) | 71.82 (11.41) |
| **Fasting blood glucose, mg/dL, mean (SD)** | 93.20 (16.00) | 98.22 (22.46) | 91.79 (13.29) |
| **Total cholesterol, mg/dL, mean (SD)** | 203.21 (36.51) | 221.55 (39.88) | 198.22 (33.85) |
| **HDL cholesterol, mg/dL, mean (SD)** | 63.92 (16.65) | 60.91 (16.97) | 64.75 (16.46) |
| **LDL cholesterol, mg/dL, mean (SD)** | 118.90 (31.45) | 134.15 (34.93) | 114.75 (29.07) |
| **Hemoglobin, g/dL, mean (SD)** | 14.30 (1.56) | 14.50 (1.56) | 14.25 (1.56) |
| **Total triglycerides, mg/dL, mean (SD)** | 101.88 (82.39) | 132.27 (111.51) | 93.54 (70.11) |
| **γ-glutamyl transpeptidase, U/L, mean (SD)** | 34.63 (41.57) | 45.53 (54.63) | 31.65 (36.65) |
| **Aspartate aminotransferase, U/L, mean (SD)** | 22.09 (11.08) | 24.22 (13.48) | 21.50 (10.25) |
| **Alanine aminotransferase, U/L, mean (SD)** | 23.16 (19.55) | 27.75 (23.70) | 21.90 (18.05) |
| **Body mass index, kg/m^2^, mean (SD)** | 22.74 (3.78) | 23.95 (4.11) | 22.41 (3.63) |

Abbreviation: LDL, low-density lipoprotein; HDL, high-density lipoprotein; SD, standard deviation.

**Table S4**. Baseline characteristics in the United Kingdom.

|  | **Total (n=408,725)** | **Dyslipidemia (n=20,719)** | **Non-dyslipidemia (n=388,006)** |
| --- | --- | --- | --- |
| **Mean age (SD), *y*** | 56.55 (8.09) | 61.30 (6.51) | 56.29 (8.08) |
| **Sex, *n (%)*** |  |  |  |
| Male | 191,445 (46.84) | 12,761 (61.59) | 178,684 (46.05) |
| Female | 217,280 (53.16) | 7,958 (38.41) | 209,322 (53.95) |
| **Region of residence, *n (%)*** |  |  |  |
| Urban | 351,026 (85.88) | 18,323 (88.44) | 332,703 (85.75) |
| Rural | 57,699 (14.12) | 2,396 (11.56) | 55,303 (14.25) |
| **Household income, *n (%)*** |  |  |  |
| Q1 (lowest income level) | 91,286 (22.33) | 7,315 (35.31) | 83,971 (21.64) |
| Q2 | 104,333 (25.53) | 6,037 (29.14) | 98,296 (25.33) |
| Q3 | 107,335 (26.26) | 4,318 (20.84) | 103,017 (26.55) |
| Q4 | 83,516 (20.43) | 2,492 (12.03) | 81,024 (20.88) |
| Q5 (highest income level) | 22,255 (5.44) | 557 (2.69) | 21,698 (5.59) |
| **Smoking status, *n (%)*** |  |  |  |
| Never smoker | 225,125 (55.08) | 9,003 (43.45) | 216,122 (55.70) |
| Ex-smoker | 141,031 (34.51) | 8,981 (43.35) | 132,050 (34.03) |
| Current smoker | 42,569 (10.42) | 2,735 (13.20) | 39,834 (10.27) |
| **Alcohol intake (days per week), n (%)** |  |  |  |
| Never | 119,963 (29.35) | 7,006 (33.81) | 112,957 (29.11) |
| 1-2 | 105,403 (25.79) | 4,821 (23.27) | 100,582 (25.92) |
| 3-4 | 97,431 (23.84) | 4,363 (21.06) | 93,068 (23.99) |
| ≥5 | 85,703 (20.97) | 4,515 (21.79) | 81,188 (20.92) |
| Unknown | 225 (0.06) | 14 (0.07) | 211 (0.05) |
| **Physical activity (sessions per week), n (%)** |  |  |  |
| 0-2 | 36,657 (8.97) | 1,842 (8.89) | 34,815 (8.97) |
| 3-4 | 65,766 (16.09) | 3,295 (15.90) | 62,471 (16.10) |
| 5-6 | 106,908 (26.16) | 5,340 (25.77) | 101,568 (26.18) |
| Every day | 185,632 (45.42) | 9,188 (44.35) | 176,444 (45.47) |
| Unknown | 13,762 (3.37) | 1,054 (5.09) | 12,708 (3.28) |
| **History of hypertension, *n (%)*** | 21,615 (5.29) | 3,386 (16.34) | 18,229 (4.70) |
| **History of diabetes, *n (%)*** | 5,852 (1.43) | 1,282 (6.19) | 4,570 (1.18) |
| **History of stroke, *n (%)*** | 1,559 (0.38) | 212 (1.02) | 1,347 (0.35) |
| **Systolic blood pressure, mmHg, mean (SD)** | 135.57 (18.59) | 141.19 (18.82) | 135.27 (18.53) |
| **Diastolic blood pressure, mmHg, mean (SD)** | 82.30 (10.28) | 83.41 (10.49) | 82.24 (10.27) |
| **Fasting blood glucose, mg/dL, mean (SD)** | 91.81 (21.36) | 99.09 (33.61) | 91.42 (20.43) |
| **Total cholesterol, mg/dL, mean (SD)** | 177.08 (35.73) | 166.29 (41.74) | 177.67 (35.28) |
| **HDL cholesterol, mg/dL, mean (SD)** | 50.53 (12.48) | 46.75 (11.77) | 50.73 (12.49) |
| **LDL cholesterol, mg/dL, mean (SD)** | 66.39 (16.63) | 62.77 (19.52) | 66.59 (16.43) |
| **Hemoglobin, g/dL, mean (SD)** | 14.20 (1.25) | 14.39 (1.25) | 14.19 (1.25) |
| **Total triglycerides, mg/dL, mean (SD)** | 114.63 (50.66) | 128.27 (55.33) | 113.89 (50.29) |
| **γ-glutamyl transpeptidase, U/L, mean (SD)** | 36.91 (40.82) | 47.39 (52.54) | 36.35 (40.03) |
| **Aspartate aminotransferase, U/L, mean (SD)** | 26.13 (10.41) | 27.95 (11.69) | 26.04 (10.33) |
| **Alanine aminotransferase, U/L, mean (SD)** | 23.52 (14.08) | 26.49 (15.81) | 23.36 (13.97) |
| **Waist circumference, cm, mean (SD)** | 90.13 (13.38) | 96.99 (13.23) | 89.76 (13.29) |

Abbreviation: LDL, low-density lipoprotein; HDL, high-density lipoprotein; SD, standard deviation.

**Table S5.** Best hyperparameter combinations for CatBoost and LightGBM.

| **Model** | **Hyperparameter** | **Value** |
| --- | --- | --- |
| **CatBoost** | random_state | 42 |
|  | bagging_temperature | 0 |
|  | border_count | 128 |
|  | colsample_bylevel | 1 |
|  | depth | 7 |
|  | iterations | 200 |
|  | l2_leaf_reg | 1 |
|  | learning_rate | 0.05 |
|  | subsample | 0.8 |
| **LightGBM** | boosting_type | gbdt |
|  | class_weight | balanced |
|  | colsample_bytree | 0.8 |
|  | importance_type | split |
|  | learning_rate | 0.1 |
|  | max_depth | 7 |
|  | min_child_samples | 20 |
|  | min_child_weight | 0.001 |
|  | min_split_gain | 0 |
|  | n_estimators | 300 |
|  | num_leaves | 31 |
|  | subsample | 0.7 |
|  | subsample_for_bin | 200000 |
|  | subsample_freq | 0 |
|  | min_data_in_leaf | 20 |

Abbreviations: CatBoost, categorical boosting; GBM, gradient boosting machine**.**

**Table S6.** Variable-wise missingness per cohort.

| **Variants** | **N** |
| --- | --- |
| Age | 0 |
| Sex | 0 |
| Region of residence | 0 |
| Household income | 0 |
| Smoking status | 6856 |
| Alcohol intake (days per week) | 22,314 |
| Physical activity (sessions per week) | 12,272 |
| History of hypertension | 0 |
| History of diabetes | 0 |
| History of stroke | 0 |
| Systolic blood pressure | 136 |
| Diastolic blood pressure | 137 |
| Fasting blood glucose | 94 |
| Total cholesterol | 134 |
| HDL cholesterol | 140 |
| LDL cholesterol | 3439 |
| Hemoglobin | 339 |
| Total triglycerides | 541 |
| γ-glutamyl transpeptidase | 92 |
| Aspartate aminotransferase | 112 |
| Alanine aminotransferase | 120 |
| Body mass index | 266 |
| Waist circumference | 314 |

Abbreviations: LDL, low-density lipoprotein; HDL, high-density lipoprotein.

**Table S7. Sensitivity analyses for model robustness and potential label leakage.**

| Data set | AUROC | Precision | Sensitivity | Specificity | Accuracy | Balanced Accuracy | F1 score | AUPRC |
| --- | --- | --- | --- | --- | --- | --- | --- | --- |
|  |  |  |  |  |  |  |  |  |
| Complete-case analysis | 0.785 | 47.0 | 50.4 | 85.5 | 78.4 | 68.0 | 48.7 | 0.469 |
| Lipid-free model† | 0.767 | 35.8 | 74.2 | 66.0 | 67.7 | 70.1 | 48.3 | 0.438 |

† Baseline LDL cholesterol, HDL cholesterol, triglyceride, and total cholesterol levels were excluded from the predictor set.

**Abbreviations:** AUROC, area under the receiver operating characteristic curve; HDL, high-density lipoprotein; LDL, low-density lipoprotein.

**Table S8.** Calibration metrics for the prediction model across cohorts.

| **Dataset** | **Calibration-in-the-large** | **Calibration slope** | **Brier Score** |
| --- | --- | --- | --- |
| South Korea | -0.63 | 1.37 | 0.15 |
| Japan | -0.65 | 1.42 | 0.17 |
| UK | -1.07 | 0.57 | 0.18 |

**Table S9.** Subdistribution hazard ratios (95% CI) for acute myocardial infarction and cerebral infarction according to tertiles of model-predicted dyslipidemia risk, estimated using Fine–Gray competing risk models.

| Probability | Model 1^*^ | Model 2^†^ |
| --- | --- | --- |
| Acute myocardial infarction |  |  |
| < Threshold | 1.0 (reference) | 1.0 (reference) |
| T1 | **0.92 (0.91 to 0.93)** | **0.99 (0.98 to 0.99)** |
| T2 | **0.91 (0.90 to 0.92)** | **0.99 (0.98 to 0.99)** |
| T3 | **0.90 (0.89 to 0.91)** | **0.97 (0.96 to 0.98)** |
| Cerebral infarction |  |  |
| < Threshold | 1.0 (reference) | 1.0 (reference) |
| T1 | **0.92 (0.91 to 0.93)** | **0.99 (0.98 to 0.99)** |
| T2 | **0.91 (0.90 to 0.92)** | 0.99 (0.99 to 1.01) |
| T3 | **0.90 (0.89 to 0.91)** | **0.98 (0.97 to 0.99)** |

Abbreviations: aHR, adjusted hazard ratio; CI, confidence interval.

^*^ Model 1: Adjusted for age and sex.

^†^ Model 2: Adjusted for age, sex, region of residence (urban and rural), household income (low [0–39 percentile], middle [40–79 percentile], and high [80–100 percentile]).

Bold indicates statistical significance (p<0.05).

The classification threshold was determined using Youden’s J statistic during five-fold cross-validation in the discovery cohort.

**Figure S1.** Overview of study cohorts and data sources.

**
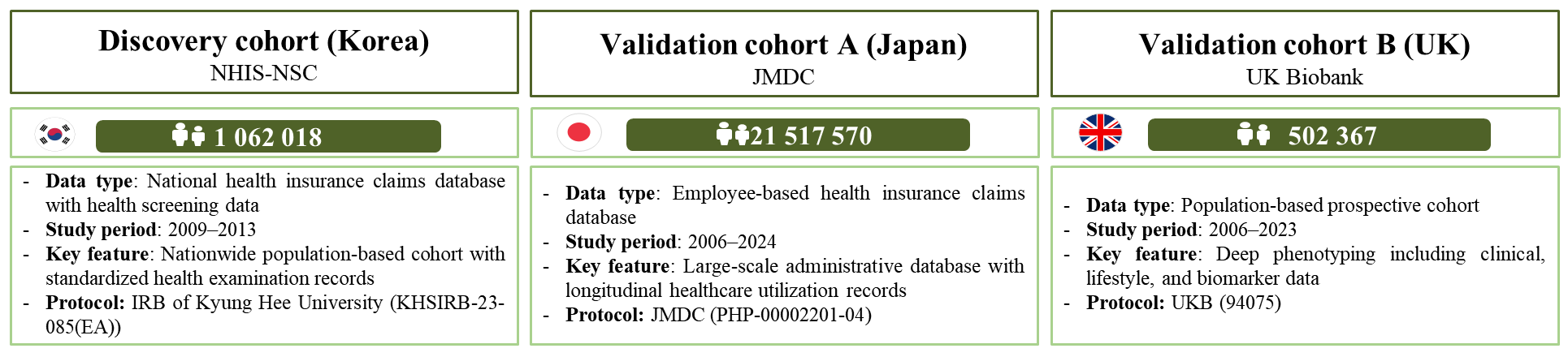
**

**Figure S2.** Calibration plot for the South Korea discovery cohort.

**
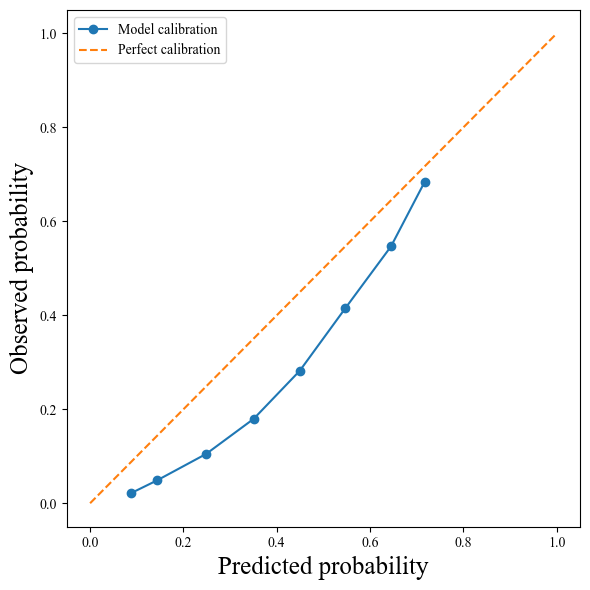
**

**Figure S3. Decision-curve analysis of the dyslipidemia prediction model in the South Korean discovery cohort.**

**
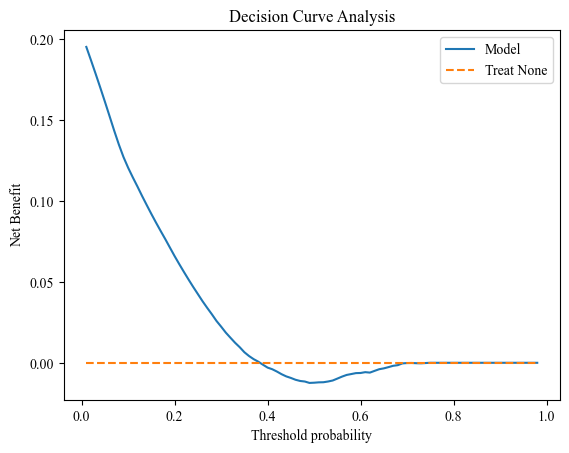
**

**Figure S4**. Web-based visualization of predicted dyslipidemia probability by site.


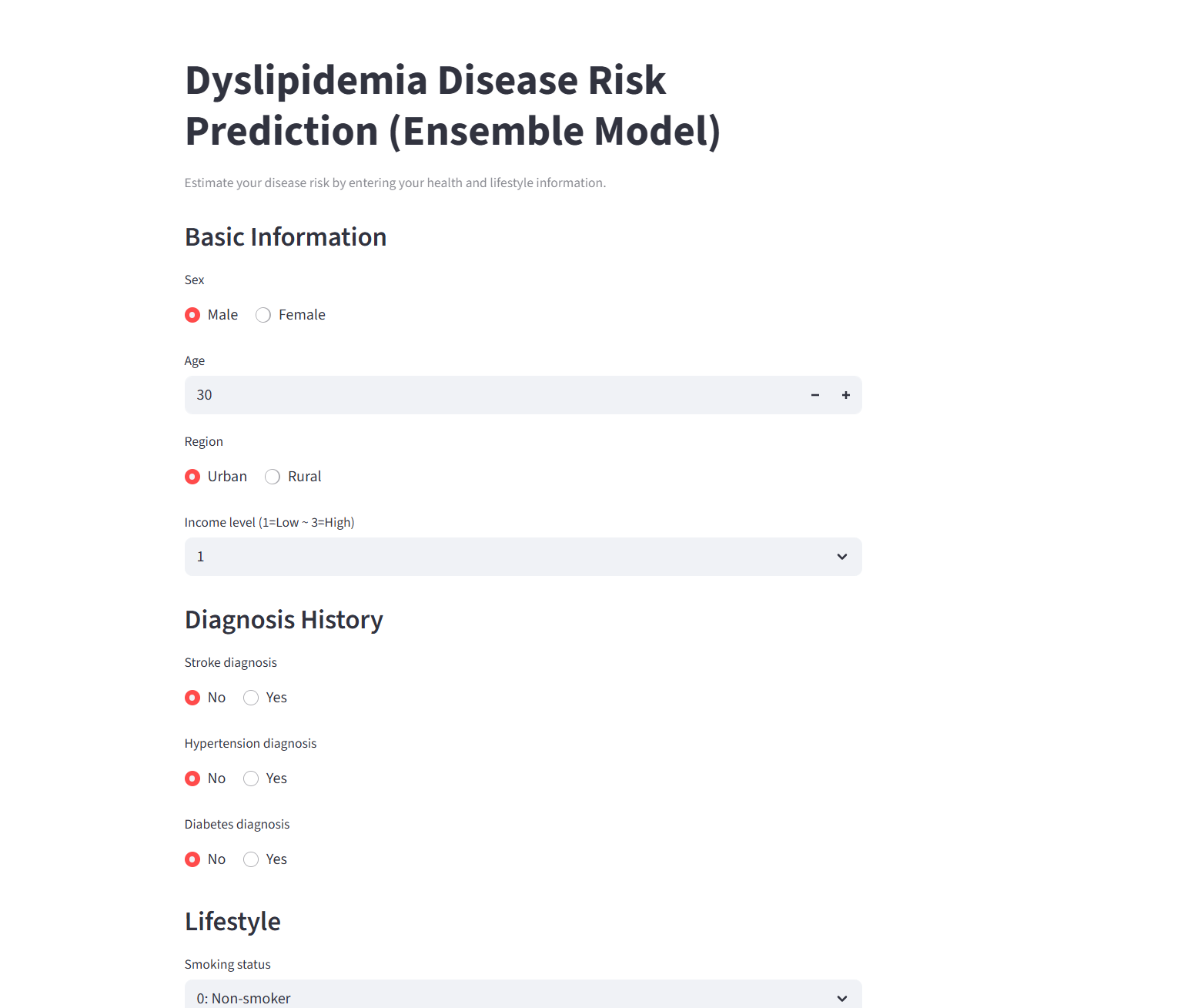

Supplement: Multimedia Appendix 1 [file medinform-v14-e81130-s001.docx]
